# Raltegravir-induced drug reaction with eosinophilia and systemic symptoms (DRESS) syndrome: implications for clinical practice and patient safety

**DOI:** 10.1186/1758-2652-13-S4-P110

**Published:** 2010-11-08

**Authors:** MEO Perry, N Almaani, N Desai, J Fox, N Larbalestier, D Chilton

**Affiliations:** 1 St Thomas' Hospital, Harrison Wing, London, UK; 2 St Thomas' Hospital, Dermatology, London, UK

## Introduction

Integrase inhibitor raltegravir is being used increasingly in patients with potential drug interactions [1]. We describe a case of DRESS syndrome in a patient who was switched to raltegravir from a PI based regime.

## Case report

A 55 yr old patient was diagnosed with HIV in 2005 with a nadir CD4 30. Virological suppression was achieved on NNRTI based HAART. Following the development of resistance this was later switched to a PI based regime with a good virological response. In order to treat her severe post herpetic neuralgia secondary to multi dermatome herpes zoster, she was given epidural corticosteroid, triamcinolone. Forty one days later she presented with Cushing's syndrome. This was due to the interaction of corticosteroid with PI.

The PI was changed to raltegravir to avoid further interactions; the patient maintained viral suppression. Four weeks after commencing raltegravir she presented with a 2-day history of a rapidly progressive generalized maculopapular rash, pruritis, malaise and pyrexia. Eosinophil count was 1.5x10^9^/l. A clinical diagnosis of DRESS syndrome was made. The timing of raltegravir initiation made it the most likely cause. Dermatologists advised treatment with emollients, topical steroid and prednisolone 30mg daily (a lower dose than usually used for DRESS syndrome, to compensate for the PI interaction). Raltegravir was stopped and PI recommenced. Skin biopsy was consistent with a drug eruption. The rash improved over the subsequent two weeks. The patient continues on a reducing steroid regime. The eosinophil count is declining.

## Conclusions

This is the first report of a severe reaction to raltegravir. DRESS syndrome is previously described in other anti retrovirals [2] but not in relation to raltegravir. Clinicians should be aware of this potential adverse event.
